# Milk Transmission of Mammalian Retroviruses

**DOI:** 10.3390/microorganisms11071777

**Published:** 2023-07-08

**Authors:** Laura M. Kemeter, Alexandra Birzer, Stefanie Heym, Andrea K. Thoma-Kress

**Affiliations:** Institute of Clinical and Molecular Virology, Friedrich-Alexander-Universität Erlangen-Nürnberg, 91054 Erlangen, Germany; laura.kemeter@uk-erlangen.de (L.M.K.); alexandra.birzer@uk-erlangen.de (A.B.); stefanie.heym@uk-erlangen.de (S.H.)

**Keywords:** retrovirus, breast milk, breastfeeding, mammalian retrovirus, oral route, virus transmission, tonsils, salivary glands, human T cell lymphotropic virus type 1 (HTLV-1), human immunodeficiency virus (HIV)

## Abstract

The transmission of viruses from one host to another typically occurs through horizontal or vertical pathways. The horizontal pathways include transmission amongst individuals, usually through bodily fluids or excretions, while vertical transmission transpires from mother to their offspring, either during pregnancy, childbirth, or breastfeeding. While there are more than 200 human pathogenic viruses to date, only a small number of them are known to be transmitted via breast milk, including cytomegalovirus (CMV), human immunodeficiency virus type 1 (HIV-1), and human T cell lymphotropic virus type 1 (HTLV-1), the latter two belonging to the family *Retroviridae*. Breast milk transmission is a common characteristic among mammalian retroviruses, but there is a lack of reports summarizing our knowledge regarding this route of transmission of mammalian retroviruses. Here, we provide an overview of the transmission of mammalian exogenous retroviruses with a focus on O*rthoretrovirinae*, and we highlight whether they have been described or suspected to be transmitted through breast milk, covering various species. We also elaborate on the production and composition of breast milk and discuss potential entry sites of exogenous mammalian retroviruses during oral transmission.

## 1. Introduction into Breast Milk Transmission of Retroviruses

The transmission of viruses between hosts is typically facilitated through horizontal or vertical pathways, the latter occurring from mothers to their offspring during pregnancy, childbirth, or breastfeeding. Although breast milk has been shown to protect newborns from several infectious diseases, few viruses are transmitted by breastfeeding [1]. Based on Koch’s postulates, five out of sixteen human viruses that had been suspected to be transmitted by breast milk fulfill the criteria of proven breast milk transmission, including human cytomegalovirus (CMV), human immunodeficiency virus (HIV), human T cell lymphotropic virus type 1 (HTLV-1), dengue virus, and Zika virus [2]. While the milk transmission of dengue and Zika virus is a matter of debate [3,4], the breast milk transmission of CMV, HIV, and HTLV-1 has been known for a longer time [5]. Both HIV and HTLV-1 are members of the family *Retroviridae*, which comprises many other retroviruses infecting various mammalian species. Yet, there is a lack of reports summarizing our knowledge about the breast milk transmission of mammalian retroviruses. Thus, this review presents a comprehensive overview of mammalian retrovirus transmission via breast milk, encompassing multiple species. Initially, the production and composition of breast milk are discussed, followed by a detailed account of mammalian exogenous retrovirus families that have been either described or suspected to be transmitted through breast milk. Moreover, we summarize an exploration of potential entry sites for mammalian retroviruses during oral transmission. 

## 2. How Is Breast Milk Produced?

To obtain a better understanding of the breast milk transmission of animal retroviruses, it is first important to comprehend lactation, the process of the production and secretion of breast milk. The production of human breast milk in response to the hormone prolactin takes place within the human mammary glands by alveolar epithelial cells, or lactocytes. These cells are organized within alveoli, which form clusters or so-called lobules. Breast milk is secreted from the lactocytes and drains from the lumen of the alveoli into a common lactiferous duct (15–20 per lactating breast) upon contraction of the myoepithelial cells lining the outside of the alveoli in a web-like pattern. The lactiferous duct transports the milk and finally opens up on the surface of the nipple, where milk can be drawn by the suckling [6]. The epithelium within the mammary gland undergoes drastic changes during the maturation of the lactating tissue as permeability declines by the closure of tight junctions within the epithelium, influencing the composition of breast milk [7,8,9]. In general, the mechanism of milk production is similar among species; however, the location and external form of the mammary glands differ extensively [10].

## 3. How Is Breast Milk Composed in Different Species?

Breast milk is a diverse and complex biological fluid system. Several factors influence its composition, which include (1) species affiliation, (2) genetics, (3) stage of lactation, (4) health status, (5) diet, (6) infant feeding, (7) term or pre-term birth, and (8) breast fullness [11,12,13,14,15,16,17,18,19,20]. With regard to the stages of lactation, breast milk can be categorized into colostrum (birth to 5 days postpartum), transitional milk (6–14 days postpartum), and mature milk (>14 days postpartum). Milk at each of these stages has its specific composition, reacting to the infant’s needs [7,21]. Therefore, maternal support for the neonatal developing immune system is most required soon after birth, whereas the nutritional demand increases over time as the infant grows. The fundamental constituents of breast milk, comprising protein, fat, lactose, oligosaccharides, cells, minerals, and water (approximately 87%), are consistent across all mammalian species [19]. Upon centrifugation, breast milk can be fractionated, resulting in a creamy top layer containing the lipid components, a cloudy supernatant, also defined as skim milk, containing whey and casein fractions, as well as a milk cell pellet [22]. Despite similar general ingredients, the composition of the solid components varies between species, with milk from ruminants differing significantly from that of non-ruminants [19]. The research on the milk composition in other species than humans and dairy animals is rather scarce but has been summarized by Park and Haenlein [23,24]. 

### 3.1. Proteins 

Among proteins, caseins are the dominant class present in milk. They include αs1-, αs2-, β-, and κ-casein and are present in self-assembled casein micelles stabilized by κ-casein [19,25]. Between distinct species, the casein-to-whey protein ratio differs between horse, donkey, goat, sheep, camel, and human milk, having a lower casein-to-whey protein ratio than cattle milk. Regarding the protein composition, human breast milk is distinguished from cow milk by the absence of β-lactoglobulin, whereas α-lactalbumin is the most abundant whey protein in human milk [19]. Other species in which low or absent levels of β-lactoglobulin have been described include camels and llamas [19,23,26]. Overall, the protein content is the highest within the first lactation stage, as the mammary gland is still permeable, and it is especially high in immunoglobulins, reflecting the high immunological need of the infant [7,8,9]. As the gut permeability of the newborn decreases soon after birth, as a result of the closure of the barrier within the epithelial tissue, the presence of maternal antibodies in the human breast milk is essential for primary mucosal immunity. This is reflected by the presence of a high proportion of soluble IgA, followed by soluble IgM and IgG [27,28,29]. Compared to this, the milk of ungulates, for instance, primarily contains IgG, while rodents transmit both IgA and IgG to the neonate [30,31]. The relevance of breast milk antibodies for gut microbiome establishment, protection against microbial and viral pathogens, allergies, and necrotizing enterocolitis has been studied in humans as well as in mouse models [28]. The two major enzymes in colostrum, lysozyme and lactoferrin, act in a cooperative manner to destroy Gram-negative bacteria; however, both exert further antibacterial, antiviral, or antifungal as well as immune-modulatory activities [29]. 

### 3.2. Fat 

The highest nutritional value within breast milk is contributed by the fat proportion, which mainly consists of triglycerides (98%), followed by diacylglycerides and free fatty acids. The triglycerides are synthesized within the rough endoplasmic reticulum of mammary epithelial cells and transported through the cytosol within the milk fat globule. Subsequently, they are secreted at the apical membrane of the mammary epithelial cell, resulting in coating by the milk fat globule membrane (MFGM), a specialized, protective phospholipid tri-layer containing specific proteins [32]. This organization of fatty components is unique to breast milk and is a common feature of all mammalian species; however, milk fat globules may vary in size and protein proportion [19]. Out of all breast milk components, the total fat composition and concentration can vary the most, not only over the course of lactation but also within one day and even within one session of nursing, leading to high differences between different individuals as well as between different species [7,19,32,33]. MFGM components, including phospholipids, gangliosides, cholesterol, and glycoproteins, and their implications on infant health regarding cognitive development, intestinal maturation, and immune development have been studied extensively and were summarized by Thum et al. [32].

### 3.3. Lactose 

Naturally and exclusively present within mammalian breast milk, lactose is the major source of carbohydrates. It is a disaccharide composed of D-glucose and D-galactose and is broken down via β-galactosidase (lactase) present in the small intestine of all mammals [34]. This enzyme reaches its peak activity shortly after birth and decreases again typically after weaning. Nevertheless, in some ethnicities in Europe and Africa, human lactase activity stays persistent even into adulthood (app. 20%), which is genetically determined [34,35,36,37]. Human milk has a relatively high lactose content (7%) compared to other species, especially compared to cow’s milk (4.6%) [19,34].

### 3.4. Oligosaccharides

Another factor significantly discriminating human breast milk from that of other mammals is the content and variability in oligosaccharides (OSs). Human milk oligosaccharides (HMOs) represent the third highest amount of solid components in human milk, and until now, over 200 different structures have been identified [38,39]. The characteristic of all HMOs is the lactose core at their reducing end, which can be extended by one or several monosaccharide structures out of galactose, N-acetylglucosamine, fucose, and N-acetylneuraminic acid. The process of HMO synthesis was summarized by Bode [38]. Nevertheless, HMOs do not serve as nutrients, as they are non-digestible by the infant and are secreted with the feces [40,41,42]. Initially, they were identified because of their prebiotic potential, as favorable gut bacteria metabolize HMOs. Therefore, they actively shape a healthy microbiome within the infant’s gut [43,44]. Furthermore, HMOs show antibiotic and antiviral functionality, as they, e.g., exhibit a decoy function for bacterial and viral receptors [38,45]. The highest concentration of HMOs is found within colostrum (20–25 g/L) and declines over time (5–20 g/L in mature milk) [38,39,40,46]. Within the human species, the OS composition varies between individuals [47,48] and is defined by genetic factors depending on the Lewis blood group antigens, dividing lactating mothers into so-called milk types (Le+/Se+, Le−/Se+, Le+/Se−, Le−/Se−). Mothers harboring an active secretor gene (Se+; >70%) express fucosyltransferase-2 (FUT2), which enables them to generate α(1-2)-fucosylated HMOs, e.g., 2′-fucosyllactose (2′-FL) and lacto-N-fucopentaose 1 (LNFP1). The milk of non-secretors (Se−) does not contain α(1-2)-fucosylated HMOs. In addition, fucosyltransferase 3 (FUT3), encoded by the *Lewis* (Le) gene, links fucose in an α1-3/4 manner, leading to a reduced amount of α1-3/4-fucosylated HMOs within Le− women [38,39,48]. 

The OS content within breastmilk has been studied in species other than humans [49,50,51,52,53,54,55]. The milk of non-human primates, for instance, still shows a more complex OS pattern than that of non-primate species [50,54]. Comparing the fucosylation status, the concentration of fucosylated OSs in chimpanzee milk (50%) is similar to that of human breast milk (50–80%), followed by gorillas (15%). In other species, the fucosylation of OSs plays a minor role (<1%) [54]. Within bovine milk, which serves as the basis of formula milk, the level of OSs is quite low [49], and porcine milk showed a highly sialylated OS pattern similar to that of bovine milk [53]. In contrast, goat milk shows significantly higher OS content (e.g., 60 to 350 mg/L in mature milk), which is still lower than that of human milk [55].

### 3.5. Cells 

Early research concluded that the human breast milk cell (hBMC) population mainly consists of leukocytes, with the majority being macrophages [56,57]. However, novel techniques such as flow cytometry and single-cell RNA sequencing (scRNA-seq) revealed that hBMC composition is more diverse and drastically changes over time [58,59,60,61]. Leukocytes, especially macrophages, are most prevalent in colostrum and early milk as the immunological need of the infant is the highest during that period, but they decline over the first two weeks postpartum to a basal level of 0–2% in mature breast milk. Therefore, luminal and myoepithelial cells are the most abundant cell subsets in mature milk. The majority of those are lactocytes—secretory epithelial cells derived from the mammary alveoli—which produce and secrete typical milk components such as HMOs, lactose, micronutrients, fat, hormones, and cytokines into breast milk [16,21,58,59,60,61]. Additionally, mesenchymal and hematopoietic stem and progenitor cells have been identified to be present in human milk [62,63,64]. Overall, the total cell number declines over time [16,65]. Despite the low basal presence of lymphocytes in mature milk, increased lymphocyte numbers could be observed after infection within the mother–infant dyad. This potentially provides the infant with additional immunological support, and baseline lymphocyte levels are restored after recovery [16,18,20]. Regarding other species, bovine milk contains macrophages, neutrophils, lymphocytes, and, different to human breast milk, only a small proportion of epithelial cells [66]. Within the dairy industry, the somatic cell count (SCC) of cattle milk is a standardized feature for quality control and udder health, as mastitis in cows is associated with an increased SCC and influences the quality of the product [67]. On the contrary, for goat milk, naturally elevated SCC resulting from apocrine secretion within their milk production has been described in the past, which does not correlate with the presence of mastitis in goats [68].

## 4. Brief Overview of Retroviruses

### 4.1. Taxonomy

Retroviruses can infect a wide range of vertebrate species, including mammals, and each subfamily has unique characteristics and pathogenic properties. The family *Retroviridae* is subdivided into two subfamilies, O*rthoretrovirinae* and *Spumaretovirinae*. While the first subfamily includes the six genera *Alpha-*, *Beta-*, *Gamma-*, *Delta-*, *Epsilonretroviruses*, and *Lentiviruses*, infecting a broad range of different species, the *Spumaretovirinae*—commonly known as foamy viruses—are subdivided into five different genera according to their host tropism, including *Bovispumaviruses*, *Equispumaviruses*, *Felispumaviruses*, *Prosimiispumaviruses*, and *Simiispumaviruses* [69]. Further, retroviruses can be divided into exogenous and endogenous retroviruses, the latter also comprising retrotransposons, which are transmitted via the germline. Exogenous retroviruses of the subfamily *Orthoretrovirinae*, the focus of this article, encode all the properties required for viral replication and the release of viral particles.

### 4.2. Retroviral Particles

**Figure 1:**
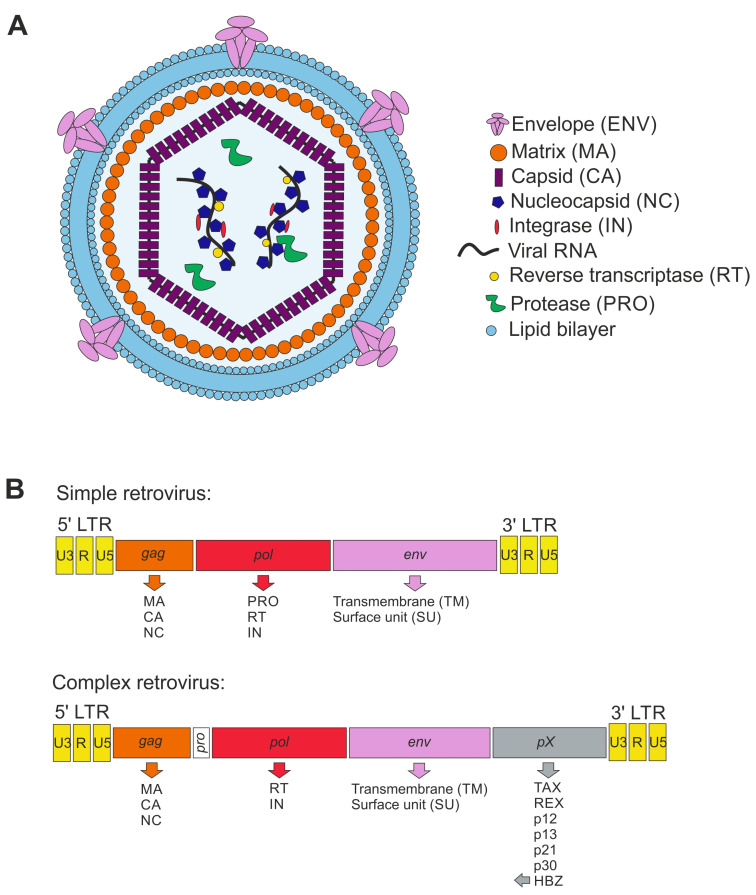
Scheme of retroviral particle and genome structure. (**A**) Retroviral particle structure enveloped by the plasma membrane as lipid bilayer containing the envelope (ENV) proteins. The matrix (MA) proteins enwrap the capsid (CA), protecting the nucleocapsid (NC) and viral RNA. Moreover, the viral proteins integrase (IN), reverse transcriptase (RT), and protease (PRO) are present in the capsid. (**B**) Schemes of integrated proviral genomes displaying simple and complex retroviruses (here: HTLV-1). The genes are shown in rectangles, and the transcribed proteins from each gene are listed below the respective gene.

Retroviral particles are around 100 nm in diameter and are enveloped with a lipid bilayer derived from the plasma membrane of the virus-producing host cell (Figure 1A). The viral envelope (ENV) proteins are usually processed by a cellular protease from a precursor protein into a transmembraneous glycoprotein (TM) and an external glycoprotein (SU). Viral matrix (MA) proteins are myristylated and line the inner surface of the viral envelope. The MA proteins surround the viral core, which is composed of viral capsid (CA) proteins. The viral capsid contains two identical copies of a single-stranded RNA, which is complexed by the nucleocapsid (NC) proteins. MA, CA, and NC are all derived from the viral gag protein precursor, which is cleaved by the viral protease during the maturation of the viral particles into the aforementioned components. Additionally, for some retroviruses such as HIV, a linker protein p6 (6 kDa) has been described, connecting CA and the viral membrane. Finally, retroviral particles contain the viral enzymes reverse transcriptase (RT), integrase (IN), and protease (PRO).

### 4.3. Genome Organization 

The capped and polyadenylated RNA genome packaged into the infectious retroviral particles is reversely transcribed by the viral RT and integrated into the host cell genome upon infection of the host target cell. The integrated proviral DNA is flanked by long terminal repeats (LTR) containing *unique 3* (U3), *redundant* (R), and *unique 5* (U5) regions, which usually harbor important regulatory sequences with promotor activity which is required for the regulation of viral gene expression (Figure 1B). Based on genome organization, retroviruses are classified into simple and complex retroviruses. Typically, simple retroviruses contain *gag*, *pol*, and *env* genes, encoding for the three group-specific antigens MA, CA, and NC (*gag*), the enzymes RT, IN, and PRO (*pol*), and ENV (*env*), respectively. Complex retroviruses additionally encode for several regulatory and accessory proteins. All human pathogenic retroviruses known to date, HIV-1, HIV-2, and the HTLVs, belong to the complex retroviruses. For more details about retrovirus organization and replication, additional reading is recommended [70,71]. 

## 5. Which Animal Retroviruses from the Family *Orthoretrovirinae* Are Transmitted by Breast Milk?

Among the animal retroviruses, *Alpharetroviruses* infecting wild and domestic birds but not mammals are excluded from this article. However, for the other five genera within the *Orthoretrovirinae*, exogenous retroviruses infecting mammals and their transmission routes, including milk transmission, are briefly described. 

### 5.1. Betaretroviruses (Non-Human)

#### 5.1.1. Jaagsiekte Sheep Retrovirus (JSRV)

JSRV (Table 1) is a *Betaretrovirus* infecting sheep and, rarely, goats [72]. It is the causative agent of ovine pulmonary adenocarcinoma, a contagious neoplastic lung disease, which leads to chronic respiratory disease, weight loss, and ultimately death [73]. Thus, JSRV belongs to the oncogenic retroviruses, and the viral ENV protein has been described as an oncogene, which is quite unique among retroviruses [74]. The tumors originate from infected type II pneumocytes and Club cells (formerly known as Clara cells), which produce pulmonary surfactant, leading to excess fluid build-up in the lungs [72]. The virus itself can be transmitted via inhalation but also through the maternal route, though details are unknown [75,76]. While JSRV mainly infects cells in the respiratory tract, infected cells can also be found in lymph nodes and white blood cells [76,77]. Interestingly, JSRV can be detected in somatic cells in colostrum and milk, for instance in milk macrophages, but not in milk lymphocytes or mammary gland epithelia [76]. Vertical transmission most likely occurs via infected cells present in colostrum and milk crossing over into the intestinal barrier of newborn lambs [76]. Since there is no vaccine available, an infection with JSRV in a flock is associated with high economic losses [78]. Recent studies also found retroviral sequences with similarity to JSRV and enzootic nasal tumor virus (ENTV; see Section 5.1.4) in dromedary camels in Saudi Arabia, which live in close proximity to sheep and goats, suggesting interspecies transmission [79]. 

#### 5.1.2. Mason–Pfizer Monkey Virus (MPMV) 

MPMV (Table 1), a rare *Betaretrovirus*, causes immunodeficiency in rhesus macaques [80,81]. This presents itself as AIDS-like symptoms, such as lymphadenopathy, weight loss, and severe opportunistic infections [82,83,84]. The transmission of the virus occurs horizontally and can be detected in saliva and milk in experimentally infected rhesus monkeys [84,85]. However, there has been no evidence of MPMV transmission via breast milk thus far.

**Table 1:** Betaretroviruses.

Virus	Natural Host	Transmission Routes	Site of Infection	Refs.
Jaagsiekte sheep retrovirus (JSRV)	Sheep, rarely goats	Inhalation of the virus;maternal route (milk)	Type II pneumocytes; somatic cells in colostrum and milk; white blood cells, lymph nodes and other lymphoid organs; bronchioalveolar epithelial cells; respiratory tract	[72,76,77,86,87,88,89]
Mason–Pfizer monkey virus (MPMV)	Rhesus macaques	Horizontal transmission	Infectious virus in saliva and milk	[80,83,84,85,90]
Mouse mammary tumor virus (MMTV)	Mice	Milk through intestinal epithelium	Dendritic cells, B lymphocytes in Peyer’s patches; T lymphocytes; epithelial tissues, including salivary gland, kidney, seminal vesicle, epididymis, and testis	[91,92,93,94,95,96,97,98,99,100]
Enzootic nasal tumor virus (ENTV)	Sheep and goats	Horizontal transmission; airborne route; nasal secretions; transuterine and oral transmission cannot be excluded	Nasal fluids; nasal epithelial cells; Bowman glands	[101,102,103,104,105,106]

#### 5.1.3. Mouse Mammary Tumor Virus (MMTV)

The most prominent *Betaretrovirus* known for its transmission via milk is MMTV (Table 1) [100,107]. Since its discovery in 1936 by John Bittner [100], the virus has been extensively studied because of its closeness to human breast cancer [108]. MMTV is not only transmitted as an exogenous retrovirus, but permanently integrated endogenous proviruses (*Mtv*) are present in most inbred mouse strains [93,109]. After the transmission of MMTV from mother to offspring, the oncogenic ‘milk factor’ can cause mammary carcinoma and lymphoma in wild and experimental mice [92,100]. The target site of the virus is the intestinal epithelium followed by epithelial tissues, including the salivary gland, kidney, seminal vesicle, and testis [92]. Following absorption through the intestine, the virus’ first target sites are dendritic cells and B lymphocytes succeeded by the activation of T lymphocytes [91,92]. Thus, the infected lymphocytes carry the virus through the lymph system into the mammary gland, causing neoplasms [95]. New evidence suggests a role of MMTV in sporadic human breast cancer, but further studies are still needed [110,111]. 

#### 5.1.4. Enzootic Nasal Tumor Virus (ENTV) 

ENTV has characteristics of both *Beta*- and *Deltaretroviruses*, but it is closely related to JSRV and infects predominantly sheep and goats (Table 1). Interestingly, ENTV is not classified by the ICTV [69]. The main route of transmission is horizontal via the airborne route and the nasal secretions contained therein [101,105]. After infection of the Bowman glands in the upper epithelial airway, ENTV can lead to respiratory signs such as dyspnea, nasal discharge and anorexia, and finally enzootic nasal adenocarcinoma [112,113]. Like JSRV, ENTV is a carcinogenic retrovirus encoding an oncogenic ENV protein. Yet, the vertical transmission of ENTV via breastmilk has not been observed, but transuterine and oral transmission cannot be fully excluded [114]. 

### 5.2. Gammaretroviruses (Non-Human)

#### 5.2.1. Feline Leukemia Virus (FeLV)

The most common cause of disease-associated death among domestic cats is attributed to the *Gammaretrovirus* FeLV (Table 2). This virus infects horizontally and vertically via several different routes of transmission [115]. The infectious virus can be found in vast quantities in saliva, feces, urine, and milk. Therefore, transmission occurs mainly through contacts between cats, such as mutual grooming and biting, the shared use of litter boxes, but also from queen to kitten while nursing via milk [115,116,117,118,119]. The initial infection starts in the mucosa of the oropharynx, leading to viral replication in the tonsils and lymph nodes. The viral spread of infected lymphocytes and monocytes through the lymphoid tissue leads to the replication of the virus in the bone marrow. This is followed by the infection of neutrophils and platelet precursors, resulting in secondary viraemia. Infected cats show clinical symptoms such as weight loss, enlarged lymph nodes, fever, neurological disorders, poor coat condition, leukemia, and in general a weak immune system [120,121,122]. Once infected, cats should be kept excluded from the outside world, especially from other FeLV-naïve cats [117,118]. A vaccine is available to prevent initial infection [123]. 

**Table 2:** Gammaretroviruses.

Virus	Natural Host	Transmission Routes	Site of Infection	Refs.
Feline leukemia virus (FeLV)	Domestic cats, cats	Horizontal transmission; friendly contacts, biting, mutual grooming, shared use of litter boxes and feeding dishes; vertical transmission; milk; from queen to kitten	Lymphocytes and monocytes in lymphoid tissue; neutrophils; saliva, feces, urine, and milk; mucosa of oropharynx; tonsils and lymph nodes; bone marrow	[115,117,118,119,120,121,122,124,125]
Gibbon ape leukemia virus (GaLV)	Captive gibbons	Transmission not fully understood	Neoplastic lymphocytic infiltration in lymph nodes, heart, liver, salivary glands, mesentery, kidney, ureters, pituitary, and choroid of eye; bone marrow	[126,127]
Koala retrovirus (KoRV)	Captive and free-living koalas	Horizontal and vertical transmission; dam–joey interactions with ingested milk, pap and/or infected fluids during perinatal period and parturition; sexual transmission not known	Unknown; no active virus recovered in milk	[126,128,129,130]
Murine leukemia virus (MLV)	Mice	Vertical and horizontal transmission; breast milk; between fighting mice	Macrophages (virus capture); T and B cells; external secretions, saliva, semen, uterine secretions; Peyer’s patch (small intestine)	[131,132,133,134]

#### 5.2.2. Gibbon Ape Leukemia Virus (GaLV) 

The infection and transmission of GaLV has been studied in captive gibbons but so far not in wild animals (Table 2). GaLV results from a transspecies transmission of a retrovirus from an unknown host, but the initial host is still not identified [126]. This virus might be able to infect non-leukemic fibroblastic cells, suggesting that the infectious virus infiltrates lymph nodes, the heart, salivary glands, liver, kidneys, ureters, pituitary, choroid of the eye, and bone marrow [126,127,135]. It can cause lymphoid tumors in sub-human primates [135]. Yet, it is unknown whether GaLV transmits via breast milk.

#### 5.2.3. Koala Retrovirus (KoRV) 

KoRV can be found in captive and wild koalas and leads to severe immunodeficiencies, usually followed by opportunistic infections such as chlamydial infections and/or neoplasias (Table 2) [126,136,137]. Thus, KoRV infection is a major threat to koalas. Like GaLV, KoRV infection results from a transspecies transmission of a retrovirus from an unknown host [126]. KoRV stands out from other retroviruses due to its distinct characteristics. While KoRV-A, one strain of the virus, is currently undergoing endogenization, KoRV-B, the main subtype, and KoRV-C, another subtype, are said to be exogenous strains [138]. Both vertical and horizontal transmission take place, but the mechanism is not fully understood [128]. Transmission might occur through dam–joey interactions, for instance through the ingestion of milk and/or infected fluids or the contact of the joey with the teat. However, no active virus has been discovered in milk thus far [128,129,130]. Certain tissue types prone to infection have not been discovered yet. 

#### 5.2.4. Murine Leukemia Virus (MLV) 

The *Gammaretrovirus* MLV (Table 2) leads to leukemia and lymphoma in mice [139]. The infectious virus is mainly found in B and T lymphocytes in external secretions, saliva, semen, and uterine secretions [131,134,139]. Transmission therefore occurs horizontally between fighting mice and vertically mainly through breast milk [131,132]. Several studies have contributed insights into the oral route of MLV transmission, which is described in Section 6.2 and Section 6.4.

### 5.3. Deltaretroviruses (Human and Non-Human)

#### 5.3.1. Bovine Leukemia Virus (BLV) 

BLV (Table 3), a virus that leads to significant economic losses in the dairy and meat industry, naturally infects cattle, water buffalo, yak, alpaca, and capybaras [140,141]. It causes enzootic bovine leukosis, presenting itself as a contagious lymphoproliferative disease of B cells [142,143,144,145]. Experimentally infected sheep serve as an animal model for the analysis of leukemia and lymphoma development upon BLV infection [146]. Transmission transpires horizontally and vertically through direct contact, nasal secretions, saliva, blood, fluids around birth, and semen. Transplacental infection and infection through insect bites have been shown as well [141,143,147,148,149,150,151,152,153]. While the presence of infectious BLV in milk and colostrum makes breast feeding a risk factor for the vertical spread of BLV, both colostrum and milk contain BLV-specific antibodies, which has been reviewed previously [141]. Although it is proven that BLV is transmitted via colostrum and milk, the initial site of infection after the ingestion of milk via the oral route is not known. Tumors associated with BLV arise mostly from various immune cell populations, such as CD5^+^/- IgM^+^ B cells, CD2^+^, CD3^+^, CD4^+^, CD8^+^ T cells, monocytes, and granulocytes in peripheral blood and lymphoid tissues [144,154,155,156,157,158]. The migration of these infected cells leads to tumors in the spleen and to a lesser extent in the liver, eye, heart, skin, lung, and lymph nodes [159,160,161,162]. Recently, a safe and effective vaccine against BLV based on an attenuated provirus has been developed, which may substitute the ‘test and eliminate’ and ‘test and segregate’ strategy [163,164]. Finally, it should be mentioned that there are controversial studies about a potential role of BLV in human breast cancer upon bovine-to-human transmission by milk or meat [165,166,167,168].

#### 5.3.2. Human T Cell Lymphotropic Virus (HTLV)

Human T cell lymphotropic virus type 1 (HTLV-1, Table 3), the only human oncogenic retrovirus, was discovered in 1981 and infects about 5–10 million people worldwide, which is probably an underestimation due to its silent transmission [169,170,171]. HTLV-1 is the causative agent of HTLV-1-associated myelopathy/ tropical spastic paraparesis (HAM/TSP), other inflammatory conditions, the incurable neoplasia adult T cell leukemia/ lymphoma (ATLL), or other adverse health outcomes [172]. Integrated HTLV-1 can predominantly be detected in CD4^+^ T cells in vivo; however, the virus is also detected in CD8^+^ T cells, dendritic cells, and monocytes, albeit at much lower levels. The transmission of HTLV-1 occurs via sexual intercourse, from mother to child, and through iaotrogenic transmission, e.g., via cell-containing blood products [173]. Mother-to-child transmission (MTCT) predominantly occurs via breastfeeding (ca. 80 % of MTCT) since abstaining from breastfeeding, or feeding for less than 90 days, can extremely reduce MTCT [174,175]. The risk factors for MTCT upon breastfeeding have been reviewed earlier, including amongst others high proviral load in milk and blood as well as prolonged breastfeeding periods for more than 6 months [176,177]. Yet, the molecular details of the oral route of HTLV-1 transmission are only partially understood, e.g., it is unknown which cells in breast milk are crucial for HTLV-1 MTCT and which cells in the infant are infected first [178]. However, abandoning breastfeeding is not an option, especially in developing countries [178]. To make matters worse, only a few countries screen pregnant women for HTLV antenatally. Finally, if HTLV-1 is transmitted via MTCT, the lifetime risk of developing ATLL rises from 5 to 20% [179]. Thus, there is a need for further research in HTLV-1 MTCT, which is also highlighted by the recent technical report of the WHO on HTLV-1 [180].

Unlike HTLV-1, the related but non-oncogenic HTLV-2 predominantly infects CD8^+^ T cells in vivo (Table 3). However, the routes of transmission are supposed to be comparable between these two viruses [181] but are less well investigated for HTLV-2 than for HTLV-1. HTLV-2 is detectable in the breast milk of infected mothers [182], but there are only a few reports on HTLV-2 MTCT by breast feeding, and comprehensive studies are lacking [183]. Although HTLV-2 is associated with increased cancer mortality, it does not induce hematologic disorders and only occasionally associates with myelopathy [184,185]. Furthermore, the two viruses display variations in their geographic distribution. HTLV-1 is widespread in Japan, sub-Saharan Africa, South America, and the Caribbean [170], whereas HTLV-2 is predominantly prevalent among indigenous populations in Africa and Indian American tribes in Central and South America, as well as among drug users in Europe and North America [184,186].

A few cases of HTLV-3 and HTLV-4 (Table 3) were reported from Cameroon [187,188]. Yet, the prevalence and transmission of these viruses are unknown, but it is estimated that zoonotic transmission of HTLV-4 may have occurred through the hunting of gorillas which were infected with the related Simian T-lymphotropic virus type 4 [189,190]. There are no data on the milk transmission of HTLV-3 or HTLV-4. 

#### 5.3.3. Simian T-Lymphotropic Virus (STLV)

The simian counterpart of HTLV-1 was discovered shortly after the human form in 1982 and belongs to the primate T lymphotropic viruses (PTLV) [191,192]. While STLV-1 (Table 3) causes ATL as its human counterpart, STLV-1 infection does not cause HAM/TSP upon infection [193]. However, the reason for this difference is still unclear. HTLV, in particular HTLV-1 and HTLV-3, appeared by cross-species transmission from monkeys to humans, which is a process still ongoing in Africa [189,194]. Based on the cross-species transmission, a high similarity of the HTLV-1/HTLV-3 and STLV-1/STLV-3 genome is given, e.g., 96% of the *Tax* sequence is identical between HTLV-1a ATK and STLV-1 *Papio anubis* strain [195,196]. While the STLV-2 strain has only been reported in wild-born bonobos in captivity, STLV-4 has been detected with PCR in gorillas in Cameroon and seems to be endemic [197]. As is known for other retroviruses, STLV is transmitted vertically from mother to child but also horizontally within the colony. MTCT in Japanese macaques in Japan was verified by Murata et al., demonstrating a correlation between aging and STLV-1 infection in the first nine years of the monkeys [198]. However, horizontal transmission occurs upon fights among monkeys as well. Moreover, due to several coinfections with other STLV strains or virus strains, vertical transmission within the colonies seems reasonable for STLV-2, STLV-3, and STLV-4. STLV-1 is the most investigated type of STLV, which was detected in the PBMCs and saliva of *Papio anubis* [199]. Recently, STLV-1 infection of neuronal cells in spinal cord regions and in brain cortical and cerebellar sections was shown [200]. 

**Table 3:** Deltaretroviruses.

Virus	Natural Host	Transmission Routes	Site of Infection	Ref.
Bovine leukemia virus (BLV)	Cattle, water buffalo, capybaras; experimental: sheep	Horizontal and vertical transmission; nasal secretions and saliva; blood; tissue; fluids around birth; colostrum and milk; transplacental; direct contact; possible transmission via insects	B lymphocytes; various immune cell populations such as CD5^+^ IgM^+^ and CD5^−^ IgM^+^ B cells; CD2^+^, CD3^+^, CD4^+^, and CD8^+^; monocytes and granulocytes in peripheral blood and lymphoid tissues	[141,143,144,147,148,149,150,151,152,153,154,155,157,158,201]
Human T-lymphotropic virus type 1 (HTLV-1)	Human	Vertical and horizontal transmission; sexual contact; contaminated blood products; breast milk	CD4^+^ and CD8^+^ T cells; dendritic cells; monocytes	[177,202,203,204,205,206,207,208,209,210]
Human T-lymphotropic virus type 2 (HTLV-2)	Human	Sexual contact; blood transfusion; breastfeeding; contaminated needles	CD8^+^ T cells	[181,186]
Human T-lymphotropic virus type 3 (HTLV-3)	Human	Unknown	PBMCs, detailed tropism unknown	[187,189]
Human T-lymphotropic virus type 4 (HTLV-4)	Human	Probably through hunting of wild gorillas	PBMCs, detailed tropism unknown	[188,189,190]
Simian T-lymphotropic virus type 1–4 (STLV-1)	Several non-human primate species, e.g., *Papio*, *Cerocebus*, and others; gorillas for STLV-4	Horizontal and maternal route; sexual contact; aggressive behavior; breastfeeding	CD4^+^ T cells; CD8^+^ T cells	[190,191,198,211,212,213,214,215,216,217,218]

### 5.4. Lentiviruses

#### 5.4.1. Bovine Immunodeficiency Virus (BIV)

BIV (Table 4), a member of the genus *Lentivirus* with a global serologic presence, infects cattle and buffalo and is transmitted vertically, in utero or transplacental, but also via blood [219]. However, BIV remains poorly understood in terms of its effects on animal health, pathogenesis, and transmission mechanisms. Yet, BIV is generally regarded as non-pathogenic and lacks any known ability to induce severe diseases in cattle [220]. Integrated proviruses have been detected in PBMCs, specifically in CD3^+^, CD4^+^, CD8^+^, and gamma-delta T cells as well as in B cells and monocytes [156]. Although BIV has been detected in the blood and milk of BIV-seropositive cows [221], there is no evidence that BIV is transmitted by milk [162].

#### 5.4.2. Equine Infectious Anemia Virus (EIAV)

EIAV (Table 4) specifically targets macrophages and results in a long-lasting infection in equids (horses, donkeys, ponies) [222]. EIAV is distributed worldwide and represents a globally significant chronic disseminated disease in veterinary medicine. EIAV gained recognition as the causative ’filterable agent’ responsible for the equine infectious anemia (EIA) syndrome in 1904, establishing one of the early instances where a viral origin was attributed to an animal disease [223]. The virus induces a persistent infection characterized by recurrent febrile episodes that involve viremia, fever, thrombocytopenia, and wasting symptoms. Among the genus *Lentivirus*, EIAV stands out due to its unique progression from a chronic stage, marked by recurring peaks of viremia and fever, to an asymptomatic stage of infection. Inapparent carriers, who show no apparent symptoms, remain infectious throughout their lives, as evidenced by experimental transmission of blood to naive animals [224]. Transmission occurs primarily through the transfer of infected blood, e.g., by biting insects such as horseflies [225]. Less common routes of transmission occur from mare to foal in utero, via milk, or sexually via semen; however, information is limited. 

#### 5.4.3. Feline Immunodeficiency Virus (FIV)

FIV (Table 4) infects domestic cats, lions, leopards, tigers, pumas, snow leopards, jaguars, Pallas’s cats, flat-headed cats, cheetahs, and bobcats. FIV is not only detectable in serum but also in blood cells, more specifically in CD4^+^ T cells, monocytes, CD8^+^ T cells, and B cells. Moreover, FIV is present in mesenteric lymph nodes, saliva, genital secretions, the brain, the thymus, bone marrow, spleen, and liver. Viruses are transmitted by biting, through sexual routes during mating (e.g., by semen), and vertically in utero and by milk [125,226,227,228,229]. The death of kittens upon in utero transmission is frequently observed. Interestingly, FIV is concentrated in milk early in lactation, and it can be experimentally transmitted via milk during acute maternal infection [230,231]. Thus, FIV provides a suitable model to study milk-borne retrovirus transmission. FIV-infected female cats bred to an FIV-negative male are widely studied, and an inactivated whole virus vaccine, Fel-O-Vax, has been developed [232].

#### 5.4.4. Caprine Arthritis Encephalitis Virus (CAEV)

CAEV (Table 4) is a small ruminant lentivirus (SRLV) that primarily affects goats and causes a multisystemic inflammatory and chronic disease known as caprine arthritis encephalitis (CAE) [233]. The virus can be transmitted vertically from infected goats to their offspring during pregnancy and postnatally via the ingestion of colostrum or milk. Another form of transfer is horizontally through long-term contact between infected animals [234,235]. CAEV infects various tissues and cells in the body, leading to arthritis and inflammation in the joints, as well as neurological symptoms such as encephalitis. The disease poses significant economic challenges as productivity decreases and veterinary costs increase [233]. Control measures such as testing, culling of infected animals, and biosecurity measures are critical to prevent the spread of CAEV in goat herds [235]. 

#### 5.4.5. Visna-Maedi Virus (VMV)

VMV (Table 4) is also an SRLV, but it infects sheep and causes Visna-Maedi (VM), a multisystemic inflammatory disease affecting various organs [236]. Like CAEV, VMV is also transmitted vertically in utero via colostrum, milk, or through the inhalation of respiratory secretions, resulting in the infection of the lungs [235,237]. Oral transmission via milk is mediated via epithelial cells in the small intestine. Infected cells can not only be found in blood, colostrum, milk, and the mammary glands but also in the lungs and semen; however, the role of the latter in transmission is unknown. The target cells of VMV are monocytes and macrophages [237]. Consequently, lesions induced by VMV are characterized by mononuclear infiltration in target organs and inflammation [17,18]. Together, SRLVs cause VM in sheep and CAE in goats, multisystemic inflammatory diseases affecting the lungs, central nervous system (CNS), joints, and mammary glands. In both cases, symptoms take several months to years to develop.

#### 5.4.6. Human Immunodeficiency Virus (HIV)

HIV-1 (Table 4), the etiologic agent of acquired immunodeficiency syndrome (AIDS), is transmitted horizontally via sexual contacts via semen, cervicovaginal, and rectal secretions, vertically from mother to child during pregnancy, child birth, or breastfeeding, and iatrogenically via blood or blood products [238]. Depending on the transmission route, different sites of entry may apply. On a cellular level, HIV infects CD4^+^ T cells or cells of the monocyte and macrophage lineage. Nowadays, sexual transmission accounts for the majority of HIV transmissions. Nevertheless, milk transmission plays an important role in MTCT and accounts for more than half of pediatric HIV infections [5,239]. Numerous studies have focused on the milk transmission of HIV and found that the risk of MTCT increases with the proviral load in breast milk, the duration of breastfeeding, and with inflammation in the mammary gland upon mastitis [239]. According to new guidelines, the WHO recommends HIV-infected mothers to receive life-long antiretroviral therapy and, under these conditions, to exclusively breastfeed for 6 months of age. Afterwards, complementary feeding should be added, but breastfeeding could continue until 24 months of age or even longer [1,240]. It is common, especially in high-income countries, to implement mixed feeding, a combination of breast milk and formula milk feeding. If mothers living with HIV are under antiretroviral therapy, the WHO states that mixed feeding is better than not to breastfeed at all [240]. However, mixed feeding is not recommended if mothers are not under therapy or adherence to antiretroviral therapy is not guaranteed since mixed feeding increases the risk of HIV transmission. Moreover, if safe infant formula or access to clean water are impaired, mixed feeding is discouraged as well since a lack of clean water, antigens in formula, and poor hygienic conditions may promote the disruption of the epithelial barrier and lead to an enhanced proinflammatory milieu in the infant, thus enhancing the risk of HIV transmission [241]. Although the treatment of the HIV-infected mother should be prioritized, routine pre-exposure prophylaxis (PrEP) of the infant should be recommended as well, especially if the mother is not treated or if maternal viral load is still detectable despite antiretroviral therapy [242]. Moreover, antiretroviral treatment of the mother does not necessarily prevent HIV cell-to-cell transmission, making routine infant PrEP even more advisable [239]. Thus, complementary actions are needed, since the risk of transmitting HIV by breastfeeding increases with maternal post-partum HIV acquisition, suboptimal adherence to antiretroviral therapy, and untreated maternal HIV infection [243].

The related HIV-2 is less pathogenic than HIV-1 and also differs in its geographic distribution since it is mainly found in West Africa, but high numbers of cases have also been found in India, Europe, and the United States. As described for HIV-1, HIV-2 is also transmitted via sexual contacts, MTCT, or iatrogenic transmission (Table 4); however, transmission rates are lower [244]. 

#### 5.4.7. Simian Immunodeficiency Virus (SIV)

SIVs are a group of viruses that infect various African primates, such as African green monkeys and sooty mangabeys. These viruses are commonly found in the wild among these primate populations but usually do not cause immunodeficiency. However, when Asian macaques are intentionally infected with these viruses in a laboratory setting, they develop a syndrome that is very similar to AIDS in humans [245]. Compared to HIV, SIV is rather transmitted by sexual routes and aggression, while mother-to-infant transmission is rare (Table 4) [245,246]. SIV infects short-lived, activated CD4^+^ T cells, monocytes, macrophages, and dendritic cells [247]. 

**Table 4:** Lentiviruses.

Virus	Natural Host	Transmission Routes	Site of Infection	Refs.
Bovine immunodeficiency virus (BIV)	Cattle and buffalo	Vertical transmission; in utero and/or transplacental; blood; exact mode unknown	Macrophages; monocytes; lymphocytes; CD3^+^, CD4^+^, and CD8^+^ T cells; B cells; milk-derived leukocytes; liver; lung; spleen; brain	[156,162,219,220,221,248]
Equine infectious anemia virus (EIAV)	Horses; donkeys susceptible	Mechanically on mouth parts of biting flies; whole blood; transplacental, colostrum, milk, semen	Monocytes; macrophages	[222,223,224,225,249,250,251]
Feline immunodeficiency virus (FIV)	Domestic cats, lions, leopards, tigers, pumas, snow leopards, jaguars, Pallas’s cats, flat-headed cats, cheetahs, and bobcats	Biting; during mating; semen; vertical transmission; milk; in utero; transmission in the wild possible through sexual routes	CD4^+^ T cells; monocytes; CD8^+^ T cells; B cells; saliva, blood, serum, plasma, genital secretions; PBMCs, brain, thymus, bone marrow, mesenteric lymph node, spleen, liver	[116,226,227,228,229,230,252,253,254]
Caprine arthritis encephalitis virus (CAEV)	Goats	Vertical transmission; colostrum; direct contact; placentas may represent transmission route; rarely sexual contact	Monocytes; macrophages; semen; genital tract; mammary gland; brain; spinal cord; lung; joints; liver; spleen; lymph node; thyroid follicle; intestinal enterocytes	[233,235,255]
Visna-maedi virus (VMV)	Sheep	Colostrum and milk; aerosol transmission; contacts between ewe and lamb, oral transmission via small intestine	Monocytes and macrophages; semen; blood; milk; colostrum; mammary gland; lung; brain	[235,236,237]
Human immunodeficiency virus 1 (HIV-1)	Human	Horizontal and vertical transmission; semen; blood and blood products; cervicovaginal and rectal secretions; maternal blood; breast milk	CD4^+^ T cells; cells of monocyte and macrophage lineage; mucosa; submucosa; draining lymphatics; gut-associated lymphoid tissue; system lymphatic tissue; vagina, ecto- and endocervix; inner foreskin; penile urethra; rectum; upper gastrointestinal tract; bloodstream	[238,256,257,258]
Human immunodeficiency virus 2 (HIV-2)	Human	Transmission similar to HIV-1; lower transmission rates	CD4^+^ T cells	[244]
Simian immunodeficiency virus (SIV)	African non-human primates	Sexual routes and aggression are suggested; rare mother-to-infant transmission	Short-lived, activated CD4^+^ T cells; monocytes; macrophages; dendritic cells	[245,246,247,259,260,261]

## 6. How Could Mammalian Retroviruses Enter Infants and Suckling Animals?

In order to develop prevention strategies for mammalian retroviral infections, it is indispensable to understand the infection routes of retroviruses. Remarkably, despite this very early and important step during viral infection, the mechanisms of oral retroviral transmission are poorly understood, especially concerning the human retroviruses. Retroviral oral transmission occurs in two different circumstances, during breastfeeding from mother to infant or via sexual contacts crossing mucosal surfaces [238,262]. One major characteristic of mammals is the feeding of their offspring via breastfeeding. During breastfeeding, the infant first gets in contact with the milk via the mouth. 

**Figure 2:**
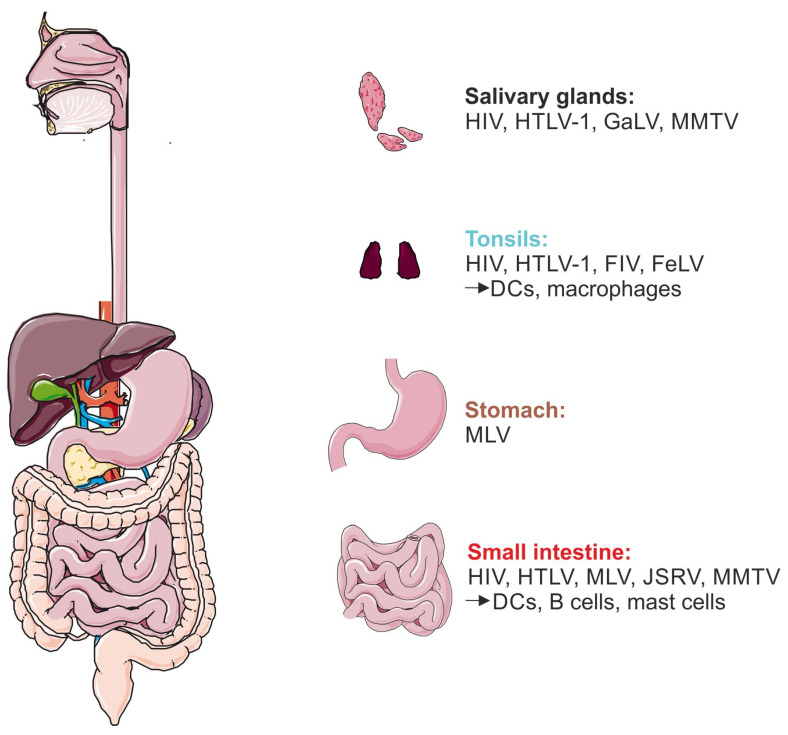
Retroviral entry sites during breast milk transmission. Possible entry sites for human and non-human retroviral infection, starting in the mouth harboring the salivary glands and tonsils, followed by the pharynx, which represents the transport path towards the stomach and small intestine. Representatively, an overview of the human digestive tract is shown. Available data on human and non-human viruses and different infected immune cells are categorized by the depicted organs. Figure created with Servier Medical Art, provided by Servier, licensed under a Creative Commons Attribution 3.0 unported license.

Observing mammals, there are six possible entry sites for mammalian retroviruses using the oral route to infect different tissues or organs containing special immune cells: (1) salivary glands, (2) tonsils, (3) the pharynx, (4) the stomach, (5) the small intestine, and (6) the colon (Figure 2). In particular, the stomach, small intestine, and colon might be difficult to pass for retroviruses since the pH value of the stomach is very acidic; for example, in Homo sapiens, the pH ranges between 1.5 and 2 [263,264]. By contrast, the tonsils but also the small intestine are inhabited by a plethora of immune cells and thus are important as entry sites.

### 6.1. Salivary Glands

The salivary glands are located within the mouth of mammals. An autoimmune disease affecting the function of salivary glands is called Sjögren’s Syndrome (SS) and is, besides other factors, associated with viral infections, in particular with HCV, EBV, HIV-1, and HTLV-1 [265,266]. The labial salivary glands of SS patients infected with HTLV-1 were positive for the *tax* but not for the *gag*, *pol*, and *env* genes [267]. Moreover, the mode of infection of salivary glands was investigated. HTLV-1 is able to infect salivary gland epithelial cells (SGECs) in SS patients but not as expected via the formation of virologic synapses but via biofilm-like structures. Thereby, the extracellular matrix proteins agrin and tetherin contributed to the infection of SGECs with HTLV-1 [268]. MMTV is a *Betaretrovirus* responsible for mammary carcinomas in mice, which is transmitted via milk to the newborn. However, MMTV has been detected in humans within the saliva and salivary glands [269]. This shows that even species-specific retroviruses can cross-infect other mammals via salivary glands as an entry site [270].

### 6.2. Tonsils

The viral transmission of retroviruses occurs via different types of transfer. The most common routes of infection with HIV occur via liquid exchange between HIV-positive patients and uninfected people via semen or vaginal fluid, saliva, blood, or breast milk [271,272]. During breastfeeding, but also during oral sex, body fluids are transferred between two organisms. Upon these activities, tonsils represent the first lymphoid organ within the oropharynx where retroviruses can enter the organism. The tonsil ex vivo model is a well-established model in the HIV research area to investigate the function of epithelial barriers upon viral infection. Using polarized tonsil epithelial cells and tonsil tissue explants, Sufiawati et al. elucidated the effect of the two HIV-1 viral proteins Tat and gp120 Env on disrupting the epithelial tight junctions to harm the barrier function of epithelial cells [273]. This clearly demonstrated that cell-free HIV-1 gp120 is able to interfere with the mucosal barrier, thus allowing HIV to overcome physical barriers in the human organism [273,274]. Tonsils are also useful to study HTLV-1 transmission, but it is unknown whether purified HTLV-1 Env has similar properties on the barrier integrity of tonsils. Although HTLV-1 infected lymphocytes failed to break the epithelial barrier using intestinal Caco-2 cells [275], it is still not settled whether the integrity of the epithelial barrier remains unaffected by HTLV-1 as well. Noteworthily, HTLV-1 has been proposed to cross the intestinal epithelial barrier by transcytosis via cell-free virions [275]. Recently, Langlois et al. showed that tonsils are efficiently infected by viral particles pseudotyped with HTLV-1 ENV ex vivo [276]. This model represents a useful tool for further ex vivo studies on tonsils infected by HTLV-1. Besides the use of pseudotyped viruses, little is known about tonsil infection with HTLV-1. A few decades ago, the tonsils of HTLV-1-infected and uninfected patients were analyzed regarding their structure. The infection with HTLV-1 implicated an atrophic change in the mantle zone of tonsils [277]. For the counterpart of HIV-1 in cats, FIV, the oral route of viral transmission was also analyzed. In oral lymphoid tissues, i.e., lymph node and tonsils, the FIV RNA was significantly higher compared to mucosal tissue, e.g., tongue and salivary gland [278]. These data hint at tonsils as a possible entry site of retroviruses. As a secondary lymphoid organ, tonsils host a plethora of immune cells. Palatine tonsils outline the first line of defense in the oropharynx, where immune defense against foreign pathogens takes place. Thus, especially T and B cells are present in tonsils; however, plasma cells and dendritic cells (DCs) were also detected [279]. Due to the unique function of DCs to present antigens, thus linking innate to adaptive immunity, DCs might play an important role in retroviral transmission by presenting (cis-infection) and transferring antigens to T cells (trans-infection) [280,281,282]. The important molecule for DC transfer to T cells is DC-SIGN. It has been shown that HTLV-1 enhances dendritic cell-specific intercellular adhesion molecule-3-grabbing non-integrin (DC-SIGN) expression on DCs, while the inhibition of this molecule interferes with the HTLV-1 infection rate [280]. The role of cis- and trans-infection was also observed for HIV-1 [283]. Dendritic cells are categorized into different subtypes: while cDC1 cells have the ability to cross-present antigens, cDC2 cells are more potent to stimulate T cells. Due to the function and presence of different subsets of DCs in the tonsils, the transfer of retroviral particles by DCs to immune cells represents a very promising early event important for the infection of mammals [279]. Besides DCs, macrophages are another important immune cell type for retroviral transmission. In the MLV mice model, sentinel macrophages capture the viral particle via CD169 receptor interaction with gangliosides, followed by subsequent trans-infection of CD19-positive B cells via synaptic contacts [284]. After passing the secondary lymphoid organ, viral transmission further invades mammals using the pharynx to reach the stomach, small intestine, and colon.

### 6.3. Pharynx

The pharynx serves as the transport path for food from the mouth to the stomach via the esophagus. Moreover, this part of the gastrointestinal tract has respiratory functions [285]. Unfortunately, data regarding the transmission of retrovirus within the pharynx are missing, although the tonsils are already located in the pharynx (see Section 6.2).

### 6.4. Gastrointestinal Tract: Stomach and Small Intestine 

Studying the gastrointestinal (GI) tract is very challenging due to the long process of digestion from the mouth to the anus. The transfer of retroviruses towards the stomach is especially difficult to analyze in experiments. However, recently, the working group from Uchil et al. and Haugh et al. established an innovative system to answer questions on virus transfer within the body. Bioluminescence imaging (BLI) enables us to track viral particles as well as to detect in vivo infection sites within the body [286,287]. Using MLV, the vertical transmission from mother to child via breastfeeding was observed in neonatal mice. Cell-free viral particles could be detected in the stomach content in the GI tract of neonatal mice, which were fed by milk from the MLV-infected mother for three days [286]. Since the pH value of the stomach is very low, e.g., in Homo sapiens 1.5 to 2 [263,264], retroviruses presumably do not survive the acidic environment. However, the effect of the acidification in the stomach on the infectivity of retroviruses is, with the exception of some older studies, largely unknown [288]. The pH value of newborns is higher compared to adults, indicating that the survival of MLV in the stomach of neonatal mice is not as challenging as in the stomach of adults [289,290]. The detection of viral components in the GI is also true for HIV-1 and HTLV-1. The viral p24 protein was present in gastric epithelium tissue samples of HIV-1-positive patients [291].

After surviving the acidic milieu in the stomach of mammals, another possible entry site for viral infection is the small intestine. The habitation of HTLV-1-infected intracellular epithelial lymphocytes, isolated from the small intestine in rabbits, supports the presence of retrovirus in gut-associated lymphoid compartments [292]. Moreover, the Peyer’s patch and mesenteric sac within the small intestine were colonized by MLV discovered by using the BLI model in mice [286]. A retrovirus of sheep, JSRV, was recently shown to be transmitted via milk to the lambs. Like MLV, JSRV was detected in the Peyer’s patch of a lamb using immunohistochemical stains of the small intestine [77]. The most prominent immune cells in the Peyer’s patch were represented by CD11c-positive DCs, followed by B cells [286]. This is also true for MMTV, since DCs and B cells in the Peyer’s patch were defined as the first target sites of this virus [91,92]. These studies signify that the Peyer’s patch within the small intestine represents an important site for retroviral infection and, thus, transmission. Jiang et al. demonstrated that another cell type, mast cells, might play a role in HIV-1 transmission [293]. Mast cells are present in the mucosa of the gastrointestinal tract and represent the main entrance of HIV-1 infection [294]. By expressing several attachment factors, HIV-1 can bind to mast cells and will be transmitted by trans-infection to CD4^+^ T cells. Regarding the human small intestine, cells were isolated from jejunal lamina propria and characterized via receptor expression, e.g., CD83, CD86, DC-SIGN, CD207, and CCR7 as jejunal DCs. Interestingly, these jejunal DCs were able to transport HIV-1 through the lamina propria mucosa for subsequent transmission into the blood [295]. 

Taken together, retroviral transmission can occur via different entry sites of mammals. Despite several studies concerning different organs and tissues infected by mammalian retrovirus (Figure 2), the final breakthrough to shed light on retroviral transmission is still missing. Consequently, investigations of the molecular mechanisms of retroviral transmission, especially for HIV-1 and HTLV-1, should be extended.

## Data Availability

Not applicable.
